# Alleviating the IL-1β-stimulated extracellular matrix degradation in osteoarthritis, and chondrocyte inflammation by *Morinda officinalis* polysaccharide via the SIRT6/NF-κB pathway

**DOI:** 10.17305/bb.2024.11437

**Published:** 2025-01-09

**Authors:** Dongfang Zhao, Shuqin Xing, Jiao Qi, Zhiqiang Wei, Jianghai Huang, Jigao Sun, Xinzhu Wen, Yafei Wang

**Affiliations:** 1 Department of Orthopaedics, Dongfang Hospital, Beijing University of Chinese Medicine, China; 2 Department of Oncology, Dongfang Hospital, Beijing University of Chinese Medicine, China; 3 Nursing in the South Hospital of Dongfang Hospital, Beijing University of Chinese Medicine, China

**Keywords:** Morinda officinalis polysaccharides, MOP, osteoarthritis, OA, sirtuin, 6 SIRT6, NF-κB pathway, extracellular matrix, ECM

## Abstract

*Morinda officinalis* polysaccharide (MOP) is a major active component of *Morinda officinalis*, known for its roles in supporting bone health and reducing oxidation and inflammation. However, no studies to date have specifically examined the effects of MOP on interleukin-1β (IL-1β)-stimulated chondrocyte inflammation or the progression of osteoarthritis (OA). To investigate, cell counting kit-8 assays were performed to evaluate MOP’s impact on the viability of human chondrocytes (C28/I2 cells). Cell damage was assessed using flow cytometry and Hoechst 33258 fluorescent staining. Inflammatory factor levels were measured via western blot and ELISA, while extracellular matrix (ECM) degradation was analyzed through immunofluorescence. The involvement of the NF-κB pathway and its regulation by Sirtuin 6 (SIRT6) were also explored using western blot. Following IL-1β treatment, C28/I2 cell viability decreased, inflammatory factor secretion increased, and ECM degradation was observed. MOP counteracted these effects by mitigating IL-1β-induced cell damage, preventing ECM degradation, and reducing inflammatory factor secretion, in a dose-dependent manner. Furthermore, IL-1β treatment suppressed SIRT6 expression, whereas MOP upregulated it. Notably, silencing SIRT6 diminished MOP’s protective effects on C28/I2 cells and reversed MOP’s suppression of the NF-κB pathway. In conclusion, MOP alleviates IL-1β-induced C28/I2 cell injury by inhibiting the NF-κB pathway through activation of SIRT6. This, in turn, reduces the inflammatory response, prevents ECM degradation, and ultimately slows OA progression.

## Introduction

Osteoarthritis (OA) is a degenerative inflammatory condition that affects the articular cartilage, subchondral bone, ligaments, joint capsule, and synovium [1, 2]. It is characterized by synovitis, degeneration of joint cartilage, and osteosclerosis, which are the primary causes of significant arthrodynia and functional impairment [3, 4]. Currently, OA affects approximately 52.5 million adult Americans, with this number projected to rise to 78.4 million by 2040 [5]. The etiology of OA is complex, involving metabolic abnormalities, diabetes, obesity, and aging [6, 7]. Previous studies have consistently demonstrated that OA is closely linked to inflammatory factors. Excessive inflammation drives the degradation of the cartilage extracellular matrix (ECM), with interleukin-1β (IL-1β)—a key pro-inflammatory factor—playing a critical role in OA progression [8, 9]. Despite the high prevalence of OA, no pharmaceutical treatments are currently approved for its therapy [10, 11]. Thus, identifying novel and effective drugs to alleviate IL-1β-stimulated chondrocyte inflammation could provide a breakthrough in OA treatment. *Morinda officinalis*, a plant of the Rubiaceae family, is widely distributed in tropical and subtropical regions. It contains numerous bioactive compounds that contribute to bone strengthening, cardiovascular protection, and anti-inflammatory and antioxidant effects [12, 13]. Among its active components, *Morinda officinalis* polysaccharide (MOP) has shown promise in improving bone density in ovariectomized rats and promoting osteogenic differentiation of bone marrow mesenchymal stem cells, suggesting potential applications in OA prevention and treatment [14, 15]. Additionally, MOP has been shown to increase tibial weight in chickens with underdeveloped tibiae, promote bone formation, and regulate calcium and phosphorus metabolism [16]. However, the effects of MOP on IL-1β-stimulated chondrocyte inflammation and OA progression remain unexplored. Sirtuin 6 (SIRT6), a member of the NAD^+^-dependent Sirtuin protein deacetylase family, plays a pivotal role in aging, cancer, inflammation, and cardiovascular diseases [17, 18]. Research by Nagai et al. [19] revealed that SIRT6 depletion in human chondrocytes exacerbates telomere dysfunction and DNA damage, leading to premature cellular senescence. Similarly, Collins et al. [20] demonstrated that cartilage-specific SIRT6 deficiency in mice worsened OA, resulting in severe subchondral bone sclerosis, cartilage damage, and osteophyte formation. These findings suggest that activating SIRT6 could be a viable therapeutic strategy for OA. Based on this evidence, we hypothesize that MOP alleviates IL-1β-induced chondrocyte damage by activating SIRT6. To test this hypothesis, we investigated the effects of MOP on IL-1β-stimulated inflammatory factor secretion in human chondrocytes (C28/I2) and ECM degradation, as well as its underlying molecular mechanisms. This study aims to elucidate the specific role of MOP in mitigating OA and to provide a theoretical foundation for its potential therapeutic application.

## Materials and methods

### Cell cultivation and treatment

C28/I2 cells, sourced from the Shanghai Cell Bank of the Chinese Academy of Sciences, were cultured in high-glucose DMEM medium (Gibco, Grand Island, NY, USA) supplemented with 1% penicillin–streptomycin (Gibco) and 10% fetal bovine serum (Gibco). The cells were incubated at 37 ^∘^C in a 5% CO_2_ environment. The culture medium was replaced every two days, and cell passaging was performed every three days. To induce inflammation, the C28/I2 cells were treated with 10 ng/mL of IL-1β (PeproTech Asia, Rocky Hill, NJ, USA) for 24 h. Following this, the cells were transfected with small-interfering RNA targeting SIRT6 (si-SIRT6) or a negative control (si-NC), both of which were obtained from Sangon Biotech (Shanghai, China). Transfection was carried out using Lipofectamine 3000 (Invitrogen, Carlsbad, CA, USA). For the subsequent cell viability assay, C28/I2 cells were co-cultured with MOP at concentrations of 10, 20, 50, or 100 µg/mL (HPLC >90%, wkq-08910, Weikeqi-biotech, Chengdu, Sichuan, China) for either 24 or 48 h. In the IL-1β+MOP group, cells were pretreated with MOP for 30 min before being exposed to IL-1β for 24 h. To further investigate whether MOP exerts its effects by modulating the NF-κB pathway, the cells were pretreated with BAY11-7082, a recognized NF-κB inhibitor (5 µM, HY-19312, MedChemExpress, Monmouth Junction, NJ, USA), for 30 min prior to modeling. MOP (50 µg/mL) was then added, and the cells were incubated for 24 h.

### Cell counting kit-8 (cck-8) assay

Initially, 1.5 × 10^ImEquation2^ C28/I2 cells were seeded into wells of a 96-well plate. After the cells adhered, the original medium was replaced with media containing varying doses of MOP. At 24 and 48 h, 100 µL of complete medium containing 10% CCK-8 reagent (Beyotime, Shanghai, China) was added to each well and incubated at 37 ^∘^C for 2 h. Following incubation, the cells were analyzed using a Varioskan LUX multimode plate reader (Thermo Fisher Scientific, Waltham, MA, USA) to measure their OD450 values.

### Flow cytometry

We conducted flow cytometry to assess cell apoptosis. C28/I2 cells in the log phase were collected and rinsed twice with PBS. Subsequently, 500 µL of binding buffer containing 5 µL of propidium iodide (Beyotime) and 5 µL of Annexin-V-FITC (HY-K1073, MedChemExpress) was added to the cells and mixed gently. The mixture was incubated at room temperature in the dark for 15 min. Finally, the samples were transferred to flow cytometry-specific tubes and analyzed using a flow cytometer within 1 h.

### Hoechst 33258 fluorescence staining

Following the method adopted by Lin et al. [21], C28/I2 cells were treated with 4% paraformaldehyde at 4 ^∘^C for a 15-min fixation, followed by three washes with PBS. Next, 100 µL of Hoechst 33258 staining solution (10 µg/mL, C1011, Beyotime) was added to the slides, which were incubated at room temperature in the dark for 10 min. After three additional PBS washes, the slides were observed under a fluorescence microscope to assess cell apoptosis.

### ELISA

Prostaglandin E2 (PGE2) ELISA kit (SEKH-0414, Solarbio, Beijing, China), IL-6 ELISA kit (PI330, Beyotime), and tumor necrosis factor-α (TNF-α) ELISA kit (PT512, Beyotime) were used to measure the secretion levels of inflammatory factors in the supernatants of C28/I2 cells. After the various treatments, C28/I2 cells were centrifuged, and the collected culture supernatant was added to the ELISA plates. Following the instructions provided with the ELISA kits, OD450 values were recorded using a microplate reader. Standard curve graphs were then generated, and the concentrations of IL-6, TNF-α, and PGE2 were calculated accordingly.

### Western blot

Proteins were extracted from C28/I2 cells using RIPA lysis buffer (Beyotime), and the protein concentration was determined using a BCA assay kit (Beyotime). Following gel electrophoresis, the protein samples were transferred to a PVDF membrane (Invitrogen) and blocked for 1 h. Subsequently, the membrane was rinsed and incubated overnight at 4 ^∘^C with primary antibodies targeting the following proteins: inducible nitric oxide synthase (iNOS, 1:1000; PA1-036, Invitrogen), cyclooxygenase-2 (COX-2, 1:1000; MA5-14568, Invitrogen), collagen type II ALPHA 1 chain (COL2A1, 1:1000; PA5-99159, Invitrogen), matrix metalloproteinase 13 (MMP13, 1:2000; PA5-27242, Invitrogen), a disintegrin and metalloproteinase with thrombospondin motifs 5 (ADAMTS5, 1:1000; PA5-14350, Invitrogen), SIRT6 (1:1000; PA5-17215, Invitrogen), IκBα (1:1000; PA5-17888, Invitrogen), phosphorylated IκBα (p-IκBα, 1:1000; MA5-15087, Invitrogen), p65 (1:500; 51-0500, Invitrogen), and phosphorylated p65 (p-p65, 1:200; ab16502, Abcam Inc., Cambridge, UK).The next day, after three washes, the membrane was incubated with a goat anti-rabbit secondary antibody (1:10,000; 31460, Invitrogen) for 1.5 h. The resulting band intensities (gray values) were analyzed using Image J software, with GAPDH (1:1000; PA1-987, Invitrogen) used as the internal reference.

### Immunofluorescence

C28/I2 cells were seeded into 12-well plates. Once the cell density reached 50%–60%, the cells were washed twice with PBS and fixed for 15 min with 4% paraformaldehyde (Beyotime) at room temperature. After fixation, the cells were permeabilized with 0.3% Triton X-100 (Sigma-Aldrich, St. Louis, MO, USA) for 10 min. Blocking was then performed with 5% bovine serum albumin (Sigma-Aldrich) for 30 min. Subsequently, the cells were incubated overnight at 4 ^∘^C with primary antibodies against COL2A1 (1:200), MMP13 (1:1000), or SIRT6 (1:200). The following day, the cells were incubated with goat anti-rabbit secondary antibody IgG (1:10,000) for 1 h at 37 ^∘^C in the dark. Finally, the nuclei were stained with DAPI (Solarbio) at room temperature in the dark for 10 min, and imaging was performed using a laser confocal microscope within 1 h.

### RT-qPCR

First, log-phase C28/I2 cells were seeded into wells of a 6-well plate. After the cells adhered to the well walls, they were treated with 1 mL of Trizol reagent (Invitrogen) to extract RNA. The RNA was carefully isolated from the cells and subsequently reverse-transcribed into cDNA using AMV reverse transcriptase (Sigma-Aldrich). Next, the synthesized cDNA was amplified using the SYBR Green qPCR mix kit (TAKARA, Tokyo, Japan). GAPDH served as an internal reference, and the relative mRNA expression levels in the cells were calculated using the 2^−ΔΔCt^ method. The sequences of the primers used were as follows: SIRT6: F: 5′-TCGACTTCCCCTGCACAATC-3′, R: 5′-TGGGGGAACTGTTTTGACCC-3′GAPDH: F: 5′-CATGTTCGTCATGGGTGTGAACC-3′, R: 5′-GGTCATGAGTCCTTCCACGATACC-3′.

### Data processing and analysis

All testings underwent three repetitions at the minimum, with results presented in form of mean ± standard deviation. Data were subjected to normalization and variance stabilization, STATA version 14 software was used for all analysis. Conduct a normal distribution and homogeneity of variance test to confirm that the data follows a normal distribution and the variance is homogeneous, and then, Student’s *t*-test and analysis of variance (ANOVA) was performed to make comparisons between different groups, with ^*^P < 0.05 implying statistical significance. After that, post-hoc comparisons were made by Bonferroni’s method. All graphs were drawn using Prism software (Graphpad 9.0).

## Results

### The outcome of MOP treatment for attenuating the IL-1β-stimulated damage of C28/I2 cells

**Figure 1.**
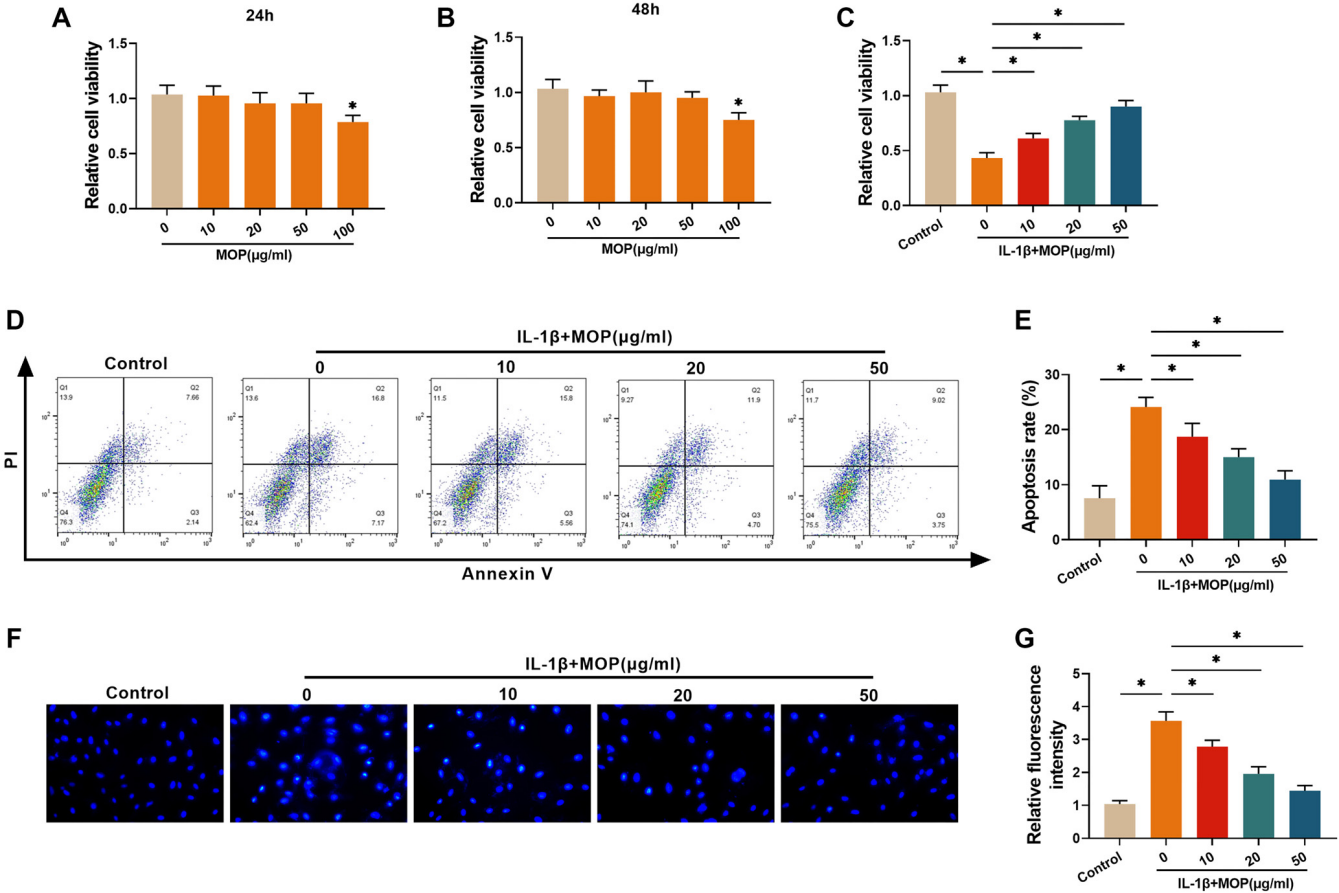
**The outcome of MOP treatment for attenuating the IL-1β-stimulated damage of C28/I2 cells.** (A and B) CCK-8 assay shows the toxicity of MOP to C28/I2 cells, demonstrating that 10, 20, and 50 µg/mL of MOP were non-toxic, while 100 µg/mL significantly reduced cell viability; (C) Viability of IL-1β-treated C28/I2 cells is markedly reduced, and MOP reverses this in a dose-dependent manner; (D and E) Flow cytometry analysis shows increased apoptosis in IL-1β-treated C28/I2 cells, which is alleviated by MOP; (F and G) Hoechst 33258 fluorescence staining demonstrates apoptotic nuclear changes (condensation and fragmentation) in IL-1β-treated cells, with MOP treatment reducing these changes. These findings suggest MOP’s dose-dependent protective effects against IL-1β-induced chondrocyte damage. ^*^P < 0.05. MOP: Morinda officinalis polysaccharide; IL-1β: Interleukin-1β; CCK-8: Cell counting kit-8.

Using the CCK-8 assay, we evaluated the viability of C28/I2 cells in response to different MOP doses (0, 10, 20, 50, and 100 µg/mL). Doses of 10, 20, and 50 µg/mL were found to be non-toxic to C28/I2 cells over 24 and 48 h of treatment, whereas cell viability decreased significantly at the 100 µg/mL dose (Figure 1A and 1B). Based on these findings, subsequent experiments were conducted using MOP doses of 10, 20, and 50 µg/mL, with a treatment duration of 24 h. Following 24 h of IL-1β treatment, the viability of C28/I2 cells was significantly reduced, but MOP treatment reversed this effect in a dose-dependent manner (Figure 1C). Furthermore, results from flow cytometry and Hoechst 33258 fluorescence staining revealed that IL-1β treatment markedly increased apoptosis in C28/I2 cells, while MOP alleviated this apoptosis (Figure 1D–1G). These findings suggest that MOP can mitigate IL-1β-induced damage to C28/I2 cells, with the extent of protection depending on the MOP dose.

### The outcome of MOP treatment for impeding the IL-1β-stimulated inflammatory mediator secretion of C28/I2 cells

**Figure 2.**
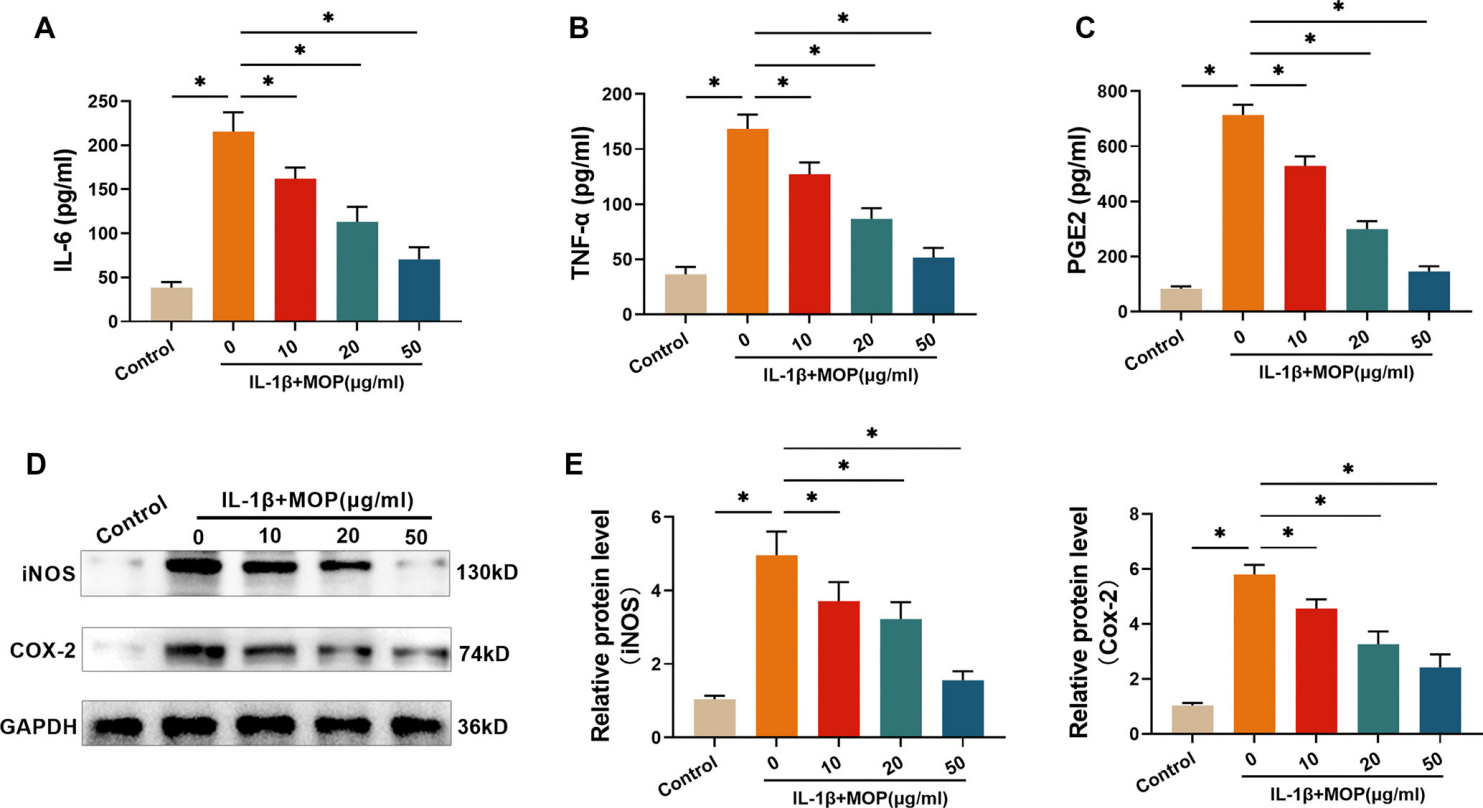
**The outcome of MOP treatment for impeding the IL-1β-stimulated inflammatory mediator secretion in C28/I2 cells.** (A–C) ELISA results show that IL-1β stimulation significantly increases PGE2, TNF-α, and IL-6 secretion in C28/I2 cells, while MOP treatment decreases these levels in a dose-dependent manner; (D and E) Western blot analysis reveals that IL-1β enhances COX-2 and iNOS protein expression, and MOP mitigates this effect. These results indicate that MOP suppresses the IL-1β-stimulated inflammatory response by reducing the secretion of key inflammatory mediators and enzyme expression. ^*^P < 0.05. MOP: Morinda officinalis polysaccharide; IL-1β: Interleukin-1β; SIRT6: Sirtuin 6; TNF-α: Tumor necrosis factor-α; PGE2: Prostaglandin E2; iNOS: Inducible nitric oxide synthase; COX-2: Cyclooxygenase-2.

PGE2, IL-6, and TNF-α levels in C28/I2 cells were measured using ELISA. The results showed that these three factors significantly increased in C28/I2 cells after IL-1β treatment (Figure 2A–2C). However, subsequent treatment with MOP (10, 20, and 50 µg/mL) effectively suppressed this increase in a dose-dependent manner. Similarly, western blot analysis revealed that COX-2 and iNOS levels were markedly elevated following IL-1β treatment (Figure 2D and 2E), but this effect was inhibited by MOP treatment. These findings suggest that MOP has a substantial ability to reduce the IL-1β-induced secretion of inflammatory mediators in C28/I2 cells.

### The outcome of MOP treatment for easing the ECM degradation in C28/I2 cells stimulated by IL-1β

**Figure 3.**
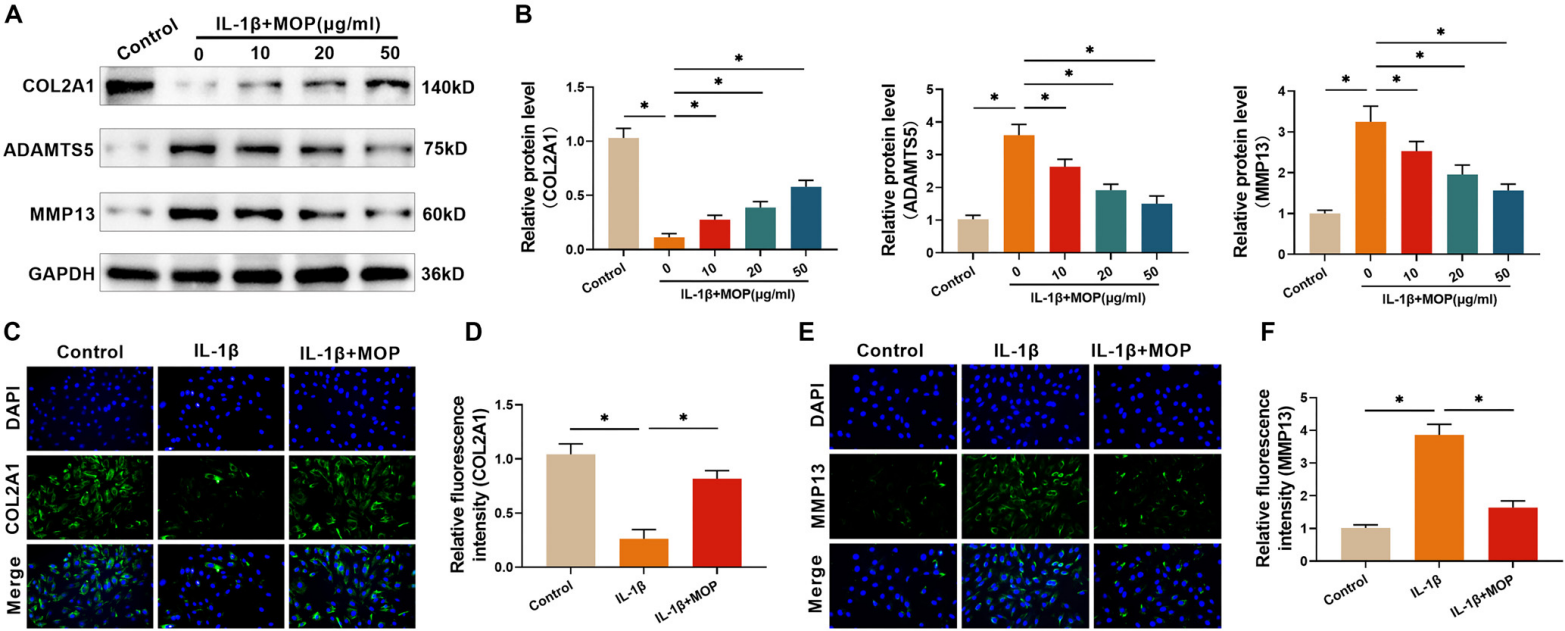
**The outcome of MOP treatment for easing the IL-1β-stimulated C28/I2 Cell ECM degradation.** (A and B) Western blot analysis shows that IL-1β stimulation reduces COL2A1 expression and increases ADAMTS5 and MMP13 levels in C28/I2 cells, while MOP treatment reverses these effects in a dose-dependent manner; (C and D) Immunofluorescence staining confirms that MOP restores COL2A1 expression in IL-1β-treated cells; (E and F) Immunofluorescence results show that MOP suppresses the IL-1β-induced increase in MMP13 levels. These findings demonstrate that MOP protects against ECM degradation induced by IL-1β. ^*^P < 0.05. MOP: Morinda officinalis polysaccharide; IL-1β: Interleukin-1β; SIRT6: Sirtuin 6; BCM: Extracellular matrix; COL2A1: Collagen type II ALPHA 1; MMP13: Matrix metalloproteinase 13; ADAMTS5: A disintegrin and metalloproteinase with thrombospondin motifs 5.

To evaluate the impact of MOP on ECM degradation induced by IL-1β, western blotting and immunofluorescence analyses were performed. The western blot results showed that IL-1β significantly suppressed COL2A1 synthesis in C28/I2 cells while increasing the protein expression levels of MMP13 and ADAMTS5. However, treatment with MOP (10, 20, and 50 µg/mL) effectively reversed these changes in a dose-dependent manner (Figure 3A and 3B). Similarly, the alterations in COL2A1 and MMP13 observed through immunofluorescence were consistent with the western blot findings. These results indicate that MOP, particularly at 50 µg/mL, can mitigate IL-1β-induced ECM degradation in C28/I2 cells (Figure 3C–3F).

### The outcome of MOP treatment for reversing the IL-1β-stimulated under-expression of SIRT6 in C28/I2 cells

**Figure 4.**
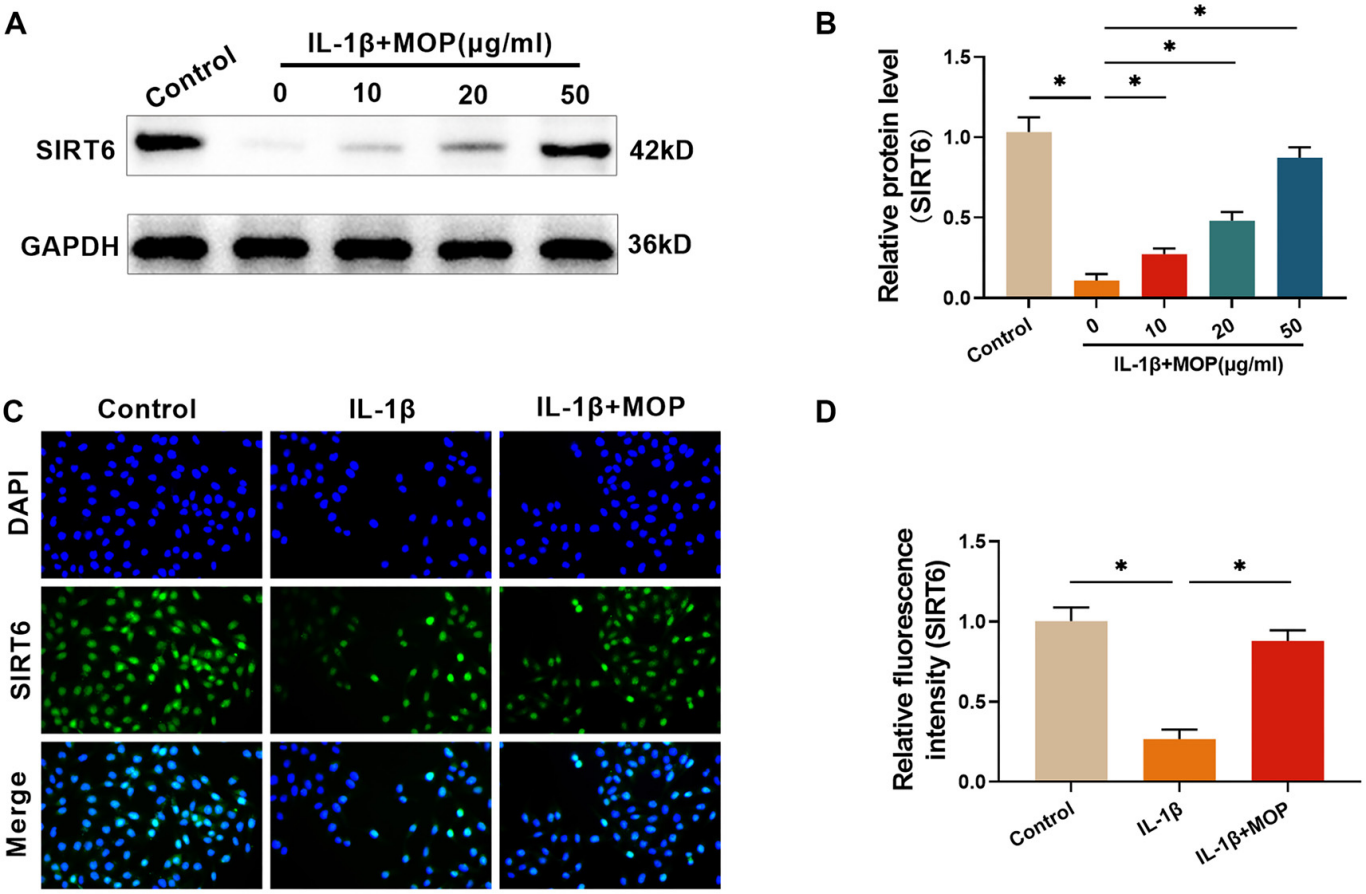
**The outcome of MOP treatment for reversing the IL-1β-stimulated SIRT6 under-expression in C28/I2 cells.** (A and B) Western blot analysis shows that IL-1β significantly reduces SIRT6 expression in C28/I2 cells, and MOP restores SIRT6 expression in a dose-dependent manner; (C and D) Immunofluorescence staining confirms MOP’s effect on SIRT6 expression, showing that MOP counteracts the IL-1β-induced reduction of SIRT6 levels. These results suggest that MOP may exert its protective effects through the upregulation of SIRT6. ^*^P < 0.05. MOP: Morinda officinalis polysaccharide; IL-1β: Interleukin-1β; SIRT6: Sirtuin 6.

Research has shown that SIRT6 overexpression can mitigate OA by alleviating chondrocyte aging and inflammation [22]. Consequently, the effect of MOP on IL-1β-stimulated SIRT6 expression in C28/I2 cells was investigated. Western blot analysis revealed a significant reduction in SIRT6 levels in C28/I2 cells following IL-1β treatment. However, MOP treatment (10, 20, and 50 µg/mL) dose-dependently increased SIRT6 levels (Figure 4A and 4B). Additionally, immunofluorescence observations indicated that MOP treatment (50 µg/mL) effectively counteracted the IL-1β-induced reduction in SIRT6 levels (Figure 4C and 4D), supporting the western blot findings. Based on these results, a concentration of 50 µg/mL was selected for subsequent experiments.

### The outcome of silencing SIRT6 for weakening the effect of MOP in improving the IL-1β-stimulated damage of C28/I2 cells

**Figure 5.**
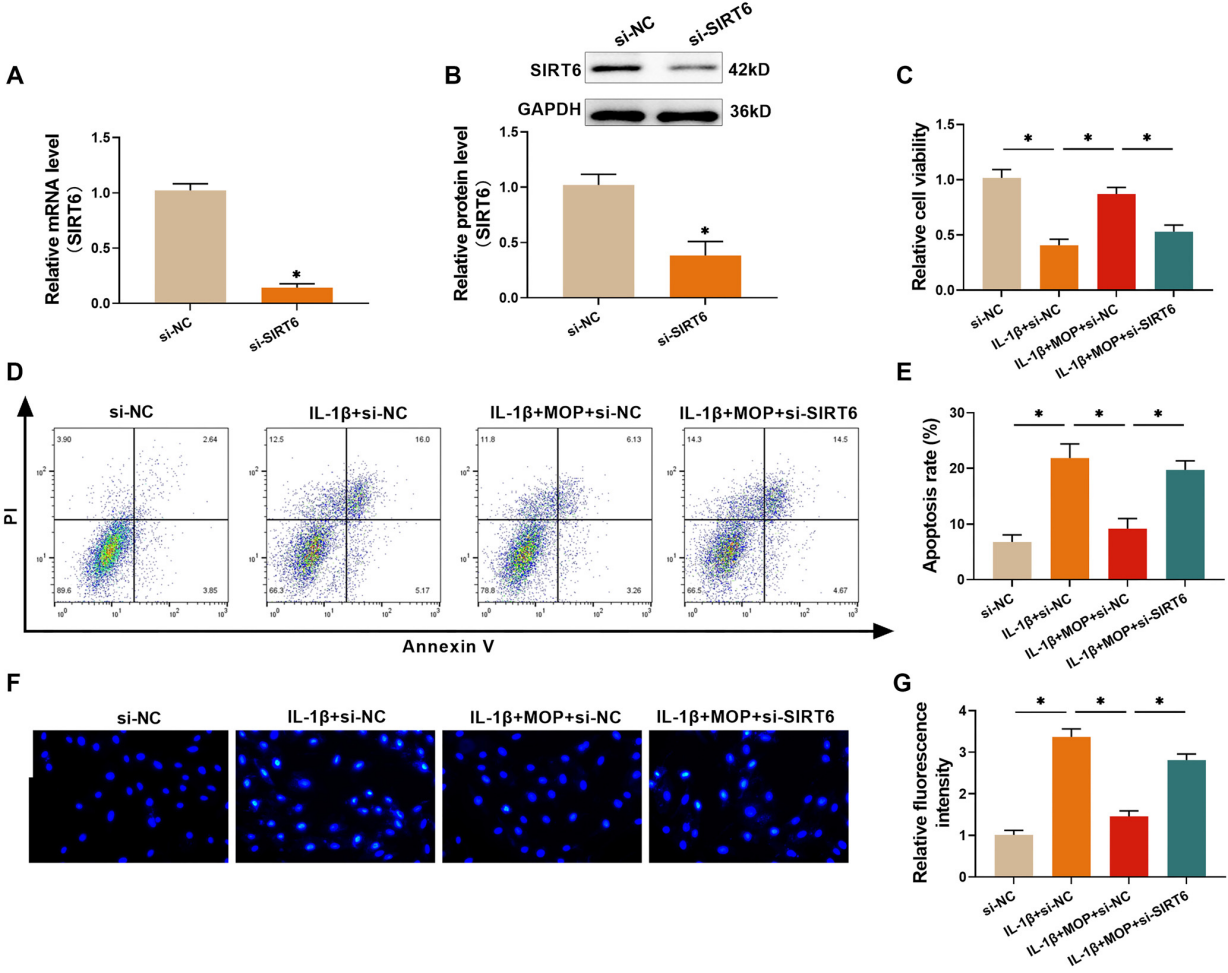
**The outcome of silencing SIRT6 for weakening the effect of MOP in improving the IL-1β-stimulateddamage of C28/I2 cells.** (A and B) Western blot and qRT-PCR analyses confirm the successful knockdown of SIRT6 via siRNA transfection in C28/I2 cells; (C) CCK-8 assay results show that the protective effect of MOP on IL-1β-treated cell viability is reduced by SIRT6 knockdown; (D and E) Flow cytometry analysis reveals that MOP reduces apoptosis in IL-1β-treated cells, but this effect is weakened when SIRT6 is knocked down; (F and G) Hoechst 33258 fluorescence staining shows that SIRT6 knockdown increases apoptosis, diminishing the anti-apoptotic effect of MOP. These results suggest that MOP’s protective effects are mediated by SIRT6. ^*^P < 0.05. MOP: Morinda officinalis polysaccharide; IL-1β: Interleukin-1β; SIRT6: Sirtuin 6; CCK-8: Cell counting kit-8.

To determine whether MOP alleviates cell damage by modulating SIRT6 levels, C28/I2 cells were transfected with si-SIRT6 and analyzed using qRT-PCR and western blot to assess SIRT6 silencing efficiency. Transfection with si-SIRT6 resulted in a sharp reduction in both SIRT6 mRNA and protein levels in C28/I2 cells (Figure 5A and 5B), indicating that the transfection efficiency met the requirements for further testing. Results from the CCK-8 assay revealed that IL-1β significantly reduced C28/I2 cell viability, while the addition of MOP (50 µg/mL) notably improved it. However, the protective effect of MOP on cell viability was diminished following SIRT6 silencing (Figure 5C). Furthermore, IL-1β promoted apoptosis in C28/I2 cells, which was mitigated by MOP. This anti-apoptotic effect of MOP, however, was weakened when SIRT6 was silenced (Figure 5D–5G). These findings suggest that MOP may alleviate IL-1β-induced damage in C28/I2 cells by regulating SIRT6 expression.

### The outcome of silencing SIRT6 for Impairing MOP’s inhibition of the IL-1β-stimulated inflammatory mediator secretion in C28/I2 cells

**Figure 6.**
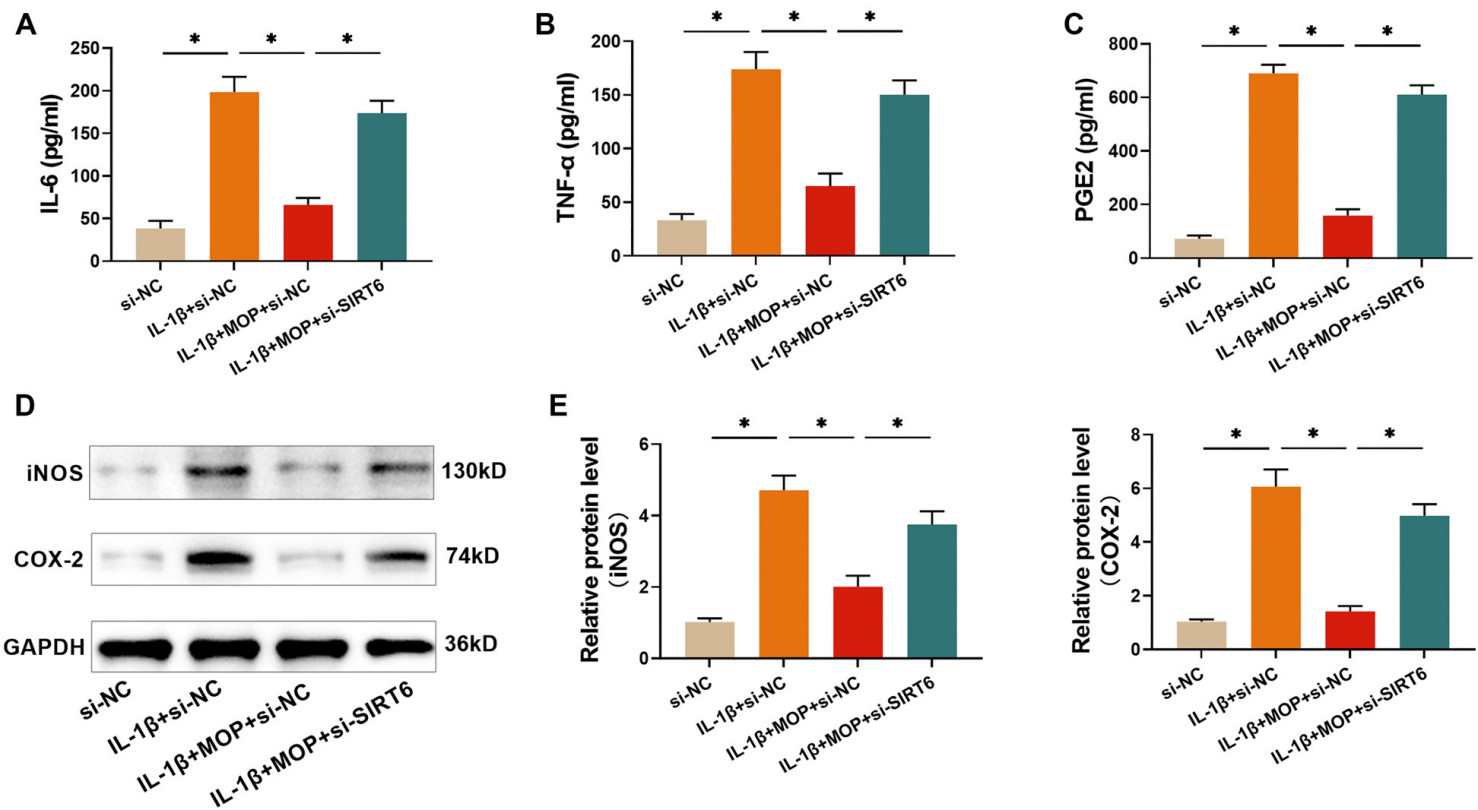
**The outcome ofsilencing SIRT6 for impairing MOP’s inhibition of the IL-1β-stimulated inflammatory mediator secretion in C28/I2 cells.** (A–C) ELISA results show that MOP inhibits the IL-1β-stimulated secretion of PGE2, TNF-α, and IL-6 in C28/I2 cells, but this effect is diminished with SIRT6 knockdown; (D and E) Western blot analysis shows that MOP reduces IL-1β-induced increases in iNOS and COX-2 levels, and SIRT6 knockdown impairs this effect. These results suggest that MOP’s anti-inflammatory effects are dependent on SIRT6 expression. ^*^P < 0.05. TNF-α: Tumor necrosis factor-α; MOP: Morinda officinalis polysaccharide; IL-1β: Interleukin-1β; SIRT6: Sirtuin 6; PGE2: Prostaglandin E2; iNOS: Inducible nitric oxide synthase; COX-2: Cyclooxygenase-2.

To investigate whether MOP exhibits anti-inflammatory effects by regulating SIRT6 expression, ELISA and western blot analyses were conducted to measure inflammatory mediator secretion in C28/I2 cells with suppressed SIRT6 expression. The results indicate that IL-1β significantly increased the secretion of PGE2, IL-6, and TNF-α in C28/I2 cells, whereas MOP (50 µg/mL) inhibited this secretion. However, silencing SIRT6 impaired the anti-inflammatory effect of MOP (Figure 6A–6C). Furthermore, IL-1β treatment led to elevated expression of iNOS and COX-2, which MOP was able to suppress. Notably, silencing SIRT6 weakened MOP’s ability to inhibit this expression (Figure 6D and 6E). These findings suggest that MOP alleviates IL-1β-induced inflammatory mediator secretion by modulating SIRT6 expression.

**Figure 7.**
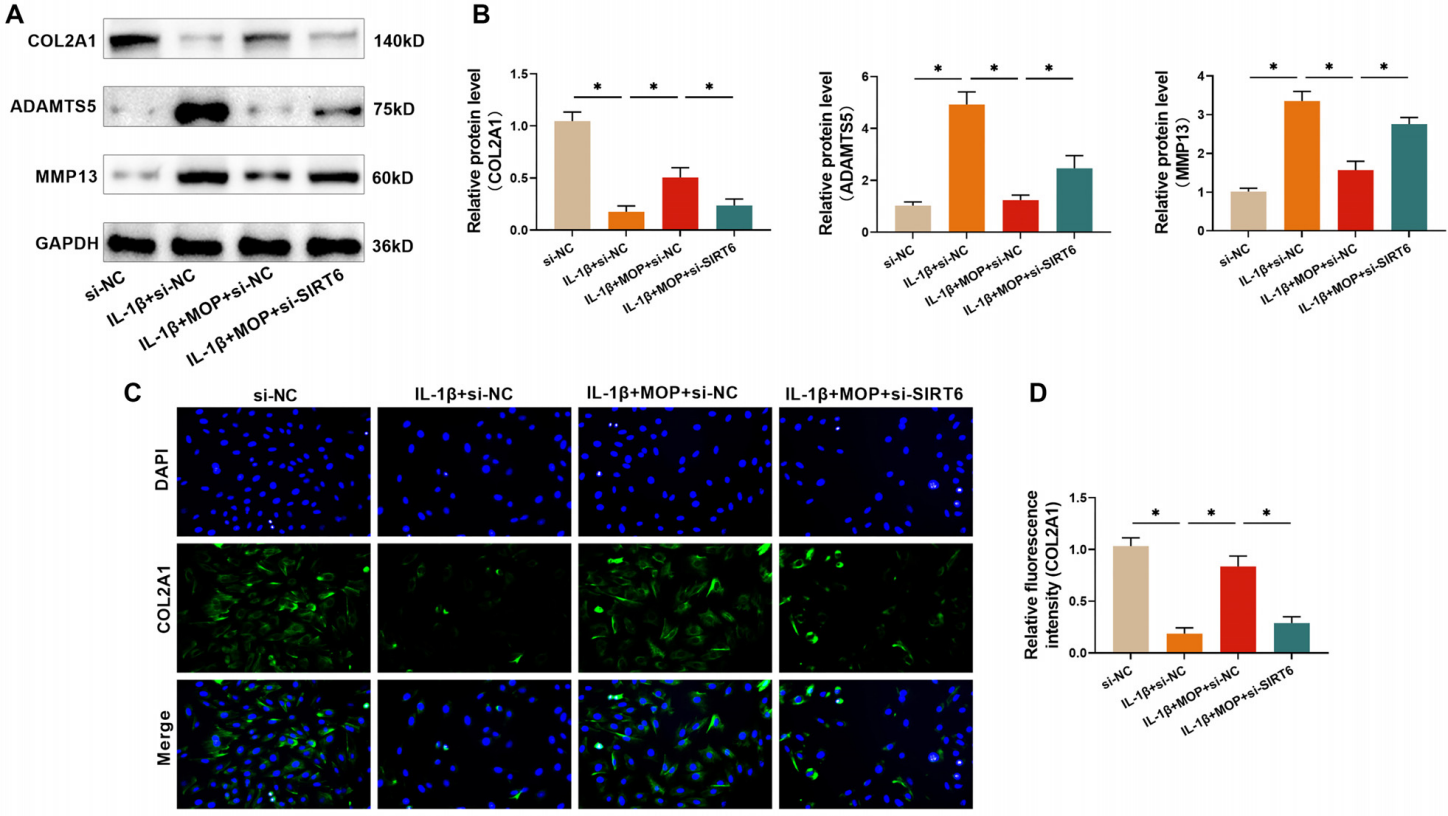
**The outcome of silencing SIRT6 for alleviating the effect of MOP in improving the IL-1β-stimulated C28/I2 cell ECM degradation.** (A and B) Western blot analysis shows that MOP reduces ADAMTS5 and MMP13 expression and restores COL2A1 levels in IL-1β-treated C28/I2 cells, but SIRT6 knockdown diminishes this effect; (C and D) Immunofluorescence staining confirms that SIRT6 knockdown reduces MOP’s ability to restore COL2A1 expression in IL-1β-treated cells. These results indicate that MOP protects against ECM degradation through a SIRT6-dependent mechanism. ^*^P < 0.05. MOP: Morinda officinalis polysaccharide; IL-1β: Interleukin-1β; SIRT6: Sirtuin 6; BCM: Extracellular matrix; COL2A1: Collagen type II ALPHA 1; MMP13: Matrix metalloproteinase 13; ADAMTS5: A disintegrin and metalloproteinase with thrombospondin motifs 5.

### The outcome of silencing SIRT6 for alleviating the effect of MOP in improving the IL-1β-stimulated C28/I2 cell ECM degradation

Western blot analysis of ADAMTS5, COL2A1, and MMP-13 expression in C28/I2 cells revealed that silencing SIRT6 significantly weakened MOP’s (50 µg/mL) protective effects against IL-1β-induced COL2A1 degradation. Additionally, SIRT6 silencing impaired MOP’s ability to suppress MMP-13 and ADAMTS5 expression (Figure 7A and 7B). Immunofluorescence analysis further confirmed that SIRT6 silencing markedly disrupted MOP’s protective effect on IL-1β-stimulated COL2A1 degradation (Figure 7C and 7D). These findings collectively demonstrate that MOP mitigates IL-1β-induced ECM degradation in C28/I2 cells by modulating SIRT6 expression.

### The outcome of MOP treatment for stimulating SIRT6 to refrain the IL-1β-stimulated NF-κB Pathway in C28/I2 cells

**Figure 8.**
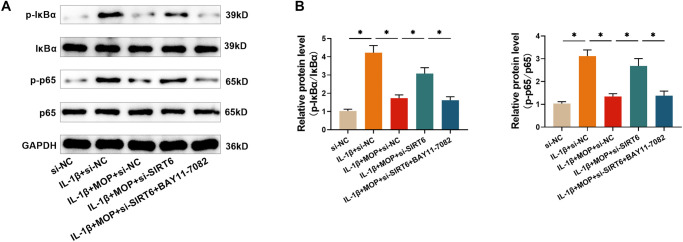
**The outcome of MOP treatment for stimulating SIRT6 to refrain the IL-1β-stimulated NF-κB pathway in C28/I2 cells.** (A and B) Western blot analysis shows that IL-1β promotes the phosphorylation of p65 and IκBα, key components of the NF-κB pathway, while MOP inhibits this phosphorylation. SIRT6 knockdown reverses MOP’s inhibitory effect on NF-κB signaling, and the NF-κB inhibitor BAY11-7082 restores the effect of MOP. These results suggest that MOP inhibits the NF-κB pathway by upregulating SIRT6, contributing to its anti-inflammatory effects in IL-1β-treated C28/I2 cells. ^*^P < 0.05. MOP: Morinda officinalis polysaccharide; IL-1β: Interleukin-1β; SIRT6: Sirtuin 6.

To further confirm the underlying mechanism, the effect of MOP on the NF-κB pathway was examined. Western blot analysis revealed that IL-1β treatment markedly promoted the phosphorylation of p65 and IκBα. Notably, this phosphorylation was significantly suppressed by MOP (50 µg/mL). However, silencing SIRT6 reversed MOP’s inhibitory effect on p65 and IκBα phosphorylation. Furthermore, BAY11-7082 (5 µM), an NF-κB inhibitor, counteracted the effects of SIRT6 silencing on MOP’s activity (Figure 8A and 8B). These findings suggest that MOP inhibits the NF-κB pathway by activating SIRT6, thereby mitigating inflammatory responses.

## Discussion

The progression of OA is widely recognized to be closely associated with the excessive secretion of inflammatory factors. Consequently, suppressing inflammation is considered a critical strategy for slowing down OA progression. A recent study has shown that *Morinda officinalis* can dose-dependently inhibit the generation of pro-inflammatory cytokines, such as iNOS and COX-2, while also preventing the nuclear translocation of NF-κB [23]. This suggests that an appropriate dose of *Morinda officinalis* may help prevent and mitigate the incidence of inflammation-related diseases. IL-1β, a key pro-inflammatory cytokine, is frequently used to induce chondrocyte inflammation as an *in vitro* model of OA [24, 25]. In this study, we observed that C28/I2 cells exhibited reduced viability following IL-1β treatment. However, MOP was able to mitigate the IL-1β-induced cell damage in a dose-dependent manner. Excessive nitric oxide (NO) production in chondrocytes can lead to cartilage destruction and cell damage, as NO synthesis in these cells is catalyzed by iNOS [26]. Similarly, TNF-α is a well-established inflammatory cytokine that plays a pivotal role in OA progression [27]. Other pro-inflammatory mediators, such as PGE2, COX-2, and IL-6, are also crucial contributors to OA pathogenesis [28, 29]. Research has demonstrated that IL-1β stimulates chondrocytes to secrete iNOS, TNF-α, and COX-2, resulting in cartilage cell dysfunction [30, 31]. Therefore, suppressing the excessive secretion of inflammatory mediators induced by IL-1β is a promising approach for OA treatment. In this study, we found that C28/I2 cells treated with IL-1β exhibited significantly increased expression of TNF-α, IL-6, COX-2, PGE2, and iNOS. Interestingly, MOP attenuated the IL-1β-induced secretion of these inflammatory mediators. These findings suggest that MOP may exert its anti-inflammatory effects by suppressing the production of these inflammatory factors.

Besides persistent inflammatory responses, the degradation of the ECM in chondrocytes is another key characteristic of OA [32]. The ECM is primarily composed of COL2A1 and aggrecan (ACAN), which provide elastic support for joint movement by dispersing pressure and shear stress—critical for maintaining the chondrocyte phenotype [33]. MMP-13 degrades COL2A1 in the cartilage ECM, while ADAMTS-5 breaks down ACAN. Excessive release of these enzymes can disrupt the ECM and lead to the loss of the normal chondrocyte phenotype [34]. In this study, IL-1β was shown to inhibit COL2A1 synthesis in C28/I2 cells while increasing ADAMTS-5 and MMP-13 protein expression, consistent with findings reported by He et al. [35]. Notably, MOP significantly reversed the IL-1β-induced downregulation of COL2A1 and the overexpression of ADAMTS-5 and MMP-13 in C28/I2 cells in a dose-dependent manner. This suggests that MOP effectively suppresses ECM degradation. In recent years, the role of SIRT6 in OA progression has gained significant attention. Liu et al. [36] found that SIRT6 mRNA levels are inversely correlated with human cartilage aging and OA severity. Similarly, Wu et al. [22] reported that chondrocytes in OA patients’ exhibit significantly lower SIRT6 levels compared to healthy individuals, and that SIRT6 overexpression can mitigate inflammatory responses and delay chondrocyte aging, thereby slowing OA progression. Moreover, studies have demonstrated that MOP improves synaptic function by modulating glucose transporter protein 3 expression, ameliorating depressive symptoms in post-stroke rats [37]. Additionally, MOP inhibits NLRP3 activation by upregulating sirtuin 1, which reduces inflammation in periodontal ligament cells [38]. Based on these findings, we hypothesized that MOP could mitigate inflammation in C28/I2 cells by regulating key transcription factors, such as SIRT6. In this study, IL-1β was observed to significantly reduce SIRT6 levels in C28/I2 cells, while MOP dose-dependently increased SIRT6 expression. Importantly, silencing SIRT6 diminished MOP’s protective effects on C28/I2 cells, indicating that MOP may alleviate IL-1β-induced ECM degradation, cell damage, and inflammation through SIRT6 modulation. MOP has also been reported to regulate various signaling pathways. For instance, it activates the PI3K/AKT pathway to promote the osteogenic and adipogenic differentiation of rat bone mesenchymal stem cells [15], and it stimulates the Wnt/β-catenin pathway to enhance the proliferation and osteogenic differentiation of bone marrow mesenchymal stem cells [39]. The NF-κB pathway, which induces PGE2, iNOS, and COX-2, is known to exacerbate joint damage by promoting tissue inflammation and chondrocyte apoptosis, making its activation a critical factor in OA progression [40]. In this study, IL-1β was shown to mediate the phosphorylation of NF-κB pathway-related proteins IκBα and p65, aligning with findings by Zhuang et al. [41]. However, MOP was found to inhibit the phosphorylation of IκBα and p65, an effect that was counteracted by SIRT6 silencing. These results suggest that MOP suppresses NF-κB pathway activation by upregulating SIRT6, which may represent a key mechanism by which MOP alleviates OA progression.This study demonstrated that MOP could mitigate IL-1β-induced inflammation and ECM degradation in human chondrocytes via the SIRT6/NF-κB pathway at the cellular model level. Future studies will focus on selecting appropriate animal models of OA to further explore and validate the mechanisms underlying MOP’s effects in a more comprehensive and detailed manner.

## Conclusion

In summary, this study demonstrated through *in vitro* cellular modeling that MOP can mitigate IL-1β-induced inflammation and damage in C28/I2 cells, as well as inhibit ECM degradation, by activating SIRT6 to suppress the NF-κB pathway. Future *in vivo* studies are required to validate the therapeutic potential of MOP in OA. Furthermore, subsequent research should explore MOP’s role in regulating SIRT6 expression in greater detail. Overall, this paper provides a new reference for the potential use of MOP in OA treatment.

## Supplemental data

**Highlights:**

1. After being treated with IL-1β, chondrocytes became less viable and secreted more inflammatory factors, along with ECM degradation.
2. MOP mitigated the IL-1β-stimulated cell damage and prevented the degradation of ECM and the secretion of inflammatory factors.
3. IL-1β dampened the expression of SIRT6, while MOP dose-dependently intensified it.
4. Silencing SIRT6 attenuated MOP’s protection for chondrocytes.
5. Silencing SIRT6 reversed MOP’s inhibition of the NF-κB pathway, while an inhibitor of this pathway neutralized this impact of SIRT6 silencing.

## Data Availability

All data used in the current study has not been deposited into a publicly available repository. In addition, the data that support the findings of this study are available from the corresponding author, [YFW], upon reasonable request.
